# Thoracolumbar Fascia Remodeling in Lumbar Spinal Stenosis: Translational Orthopedic Perspectives

**DOI:** 10.3390/ijms27198562

**Published:** 2026-09-25

**Authors:** Caterina Fede, Clara De Luca, Claudia Clair, Noa Martonovich, Andrea Angelini, Pietro Ruggieri, Carla Stecco

**Affiliations:** 1 Institute of Human Anatomy, Department of Neuroscience, University of Padova, 35122 Padova, Italy; clara.deluca@unipd.it (C.D.L.); claudia.clair@unipd.it (C.C.); carla.stecco@unipd.it (C.S.); 2 Department of Orthopedic Surgery, Hillel Yaffe Medical Center, Hadera 3820302, Israel; noamartinowitch@gmail.com; 3 Rappaport Faculty of Medicine, Technion Israel Institute of Technology, Haifa 3200003, Israel; 4 Department of Orthopedics and Orthopedic Oncology, University of Padova, 35128 Padova, Italy; andrea.angelini@unipd.it (A.A.); pietro.ruggieri@unipd.it (P.R.)

**Keywords:** lumbar spinal stenosis, thoracolumbar fascia, fibrosis, collagen, inflammation, MMP-2

## Abstract

Lumbar spinal stenosis (LSS) is a major cause of pain and disability, yet its pathophysiology remains incompletely understood. Although spinal decompression is the standard surgical treatment for LSS with disabling pain or with progressive neurological symptoms, clinical outcomes are often suboptimal, with reoperation required in 5–23% of cases. While degenerative changes in the vertebral canal have been extensively investigated, the potential contribution of the thoracolumbar fascia (TLF) has never been explored at the molecular level. We hypothesized that TLF undergoes extracellular matrix remodeling and inflammatory changes in LSS. Thoracolumbar fascia biopsies were collected from 14 patients undergoing spinal surgery (seven with LSS and seven controls with traumatic vertebral fractures). Total collagen content was quantified by hydroxyproline assay; collagen subtypes (I, III, VI and XII) by Western blotting; and inflammatory activity and extracellular matrix remodeling by ELISA quantification of TNF-α and MMP-2. LSS patients showed significantly higher hydroxyproline content (*p* = 0.026), indicating increased collagen deposition and fibrotic fascial remodeling, together with elevated MMP-2 levels (*p* = 0.038), consistent with active chronic extracellular matrix remodeling. TNF-α levels did not differ significantly between groups, although marked inter-individual variability was observed. Collagen subtype expression showed distinct trends but no significant differences. These findings provide the first molecular evidence of fibrotic remodeling and altered extracellular matrix turnover in TLF of LSS patients, supporting its role as an active contributor to disease pathophysiology and a potential diagnostic biomarker and therapeutic target.

## 1. Introduction

Lumbar spinal stenosis (LSS) is, together with lumbar strain, sprain, degenerative processes of disk and facets, disk herniation, and osteoporotic vertebral fractures, one of the mechanical causes of low back pain, which is a world-wide high-burden medical condition affecting about 40% of the population at some point in their life [1,2,3,4,5,6]. Typical symptoms of LSS are spinal claudication, paresthesia and cramping of one or both the lower extremities [4,7]. The degenerative changes leading to LSS usually involve the facet joints, ligamentum flavum, vertebral body and intervertebral disk [1,2,3,4], but despite extensive investigation of osseous, discal and ligamentous degeneration, these structural abnormalities do not fully explain symptom severity or clinical outcome. Indeed it is well known that mild-to-moderate stenosis can also be found in asymptomatic individuals [8,9]. In addition, although if spinal decompression is the surgical treatment of choice for LSS associated with disabling pain despite non-surgical interventions or with progressive neurological symptoms [10,11,12,13], the literature evidence suggests that the surgical management does not always produce the expected outcome, and in 5–23% of cases reoperation is required [7,14,15,16,17,18]. Moreover, several studies report that the risk of recurrent symptoms during follow-up time at the same level of the original disease is between 10 and 25% [8,15,16,19]. Several studies showed that the benefits of surgical over non-surgical interventions tend to diminish over time [9,18,19,20]. In addition to this, the Degenerative Lumbar Spinal Stenosis Work Group of the North American Spine Society’s (NASS) disclosed a consensus statement in 2013 observing that some of the medical treatments used for the treatment of LSS do not significantly change the course of the disease, and the evidence available did not allow them to recommend any specific pharmacological treatment [4]. A recent review concluded that the comparative efficacy between surgical and conservative treatments for LSS is not well established, due to the low quality of available evidence and overlapping long-term results [9]. Moreover, Minamide et al. investigated the clinical outcomes in a cohort of patients with LSS treated conservatively; at 10-year follow-up, approximately 30% of patients showed improvement of symptoms, 30% of patients had their symptoms unchanged and 30% showed worsening of symptoms [21].

Bearing this evidence in mind, it is therefore clear that LSS should be further investigated to identify other factors involved in its etiopathogenesis. The thoracolumbar fascia has recently emerged as a biologically active connective tissue involved in spinal biomechanics, force transmission and nociception [22,23,24,25,26,27]. In addition, increasing imaging evidence highlights that TLF undergoes structural alterations in patients with chronic low back pain, such as increased thickness, reduced shear strain, and altered mobility [26,28,29,30,31,32,33,34]. For instance, Pirri et al. showed by US that the thickness of TLF at the level of L3 is significantly greater in patients affected by non-specific LBP compared to healthy controls [35]; Langevin et al. demonstrated reduced TLF mobility in patients with LBP [36] and Tomita et al. showed, via quantitative US, microstructural alterations in TLF of individuals affected by non-specific LBP compared to healthy individuals [37]. However, Tomita et al. also observed opposite behavior of TLF, as they measured a greater shear strain of TLF in individuals affected by non-specific LBP compared to controls [38]. However, current evidence relies almost exclusively on imaging findings [39,40,41,42], while the molecular composition of the thoracolumbar fascia in lumbar spinal stenosis remains completely unexplored. We hypothesized that TLF from patients with LSS exhibits molecular features consistent with fibrotic and inflammatory remodeling. Therefore, the aim of this study was to characterize extracellular matrix remodeling and inflammatory markers in the thoracolumbar fascia of patients undergoing surgery for LSS, and to compare these findings with samples obtained from a control group of healthy patients undergoing surgery for vertebral traumatic events.

## 2. Results

Between January 2025 and November 2025, 14 patients scheduled for spine surgery as part of the treatment for LSS or vertebral fracture in the Orthopaedic Clinic of the University of Padua were selected to participate to the present study according to previously established inclusion criteria. Among them, seven patients were diagnosed with LSS and seven patients had a diagnosis of vertebral fracture with surgical indication. There were four female participants, while there were 10 male participants. Among the patients with LSS, three were female and four were male; in the control group, one patient was female and six were male. Average ODI score previous to surgery in the patients suffering from LSS was 48.8%, meaning severe disability [43]. All demographic and clinical data, including sex, age, BMI, and ODI, are reported in Table 1. No statistically significant differences were observed between the two groups in age (*p* = 0.073) or BMI (*p* = 0.053).

### 2.1. Collagen Content Analysis

Hydroxyproline was investigated as an indication of the collagen content of the samples; the average content of hydroxyproline was 0.7 ± 0.19 μg/mg in the *cases* group and 0.43 ± 0.15 μg/mg in the control group (*p* = 0.0262), with variability among LSS patients (Figure 1). This difference remained statistically significant after adjustment for age and BMI using ANCOVA (*p* = 0.039).

In the overall cohort, hydroxyproline content showed a mild positive linear association with age (r=0.38), indicating a slight trend toward increased hydroxyproline content with advancing age, although this association did not reach statistical significance (*p* = 0.18, Figure 2A). When the analysis was stratified by group, the control group showed a nearly flat regression line (r=0.10, p=0.72, Figure 2B), whereas the LSS group demonstrated higher baseline hydroxyproline levels across the entire age range, with negligible correlation with age (*r* = 0.15, *p* = 0.61, Figure 2C).

Regarding BMI, no relevant linear association was observed in the overall sample (r=0.14, *p* = 0.62, Figure 2D) or in the control group (*r* = 0.14, *p* = 0.64, Figure 2E). In contrast, the LSS group showed a moderate negative linear correlation between BMI and hydroxyproline content (*r* = −0.55, *p* = 0.04, Figure 2F), indicating lower hydroxyproline content with increasing BMI in this subgroup.

Importantly, hydroxyproline levels were consistently higher in the LSS group than in controls across the observed BMI and age ranges, suggesting that the observed differences are more likely related to the presence of lumbar spinal stenosis rather than to age or BMI alone.

Moreover, a moderate inverse linear association was observed between ODI score and hydroxyproline content in the thoracolumbar fascia of patients with LSS (*r* = −0.51, *p* = 0.06, Figure 3). However, the wide dispersion of the data points indicated considerable variability in collagen levels across clinical disability.

The immunoblot analysis revealed that the normalized content of Collagen I was 0.12 ± 0.11 in the stenosis group and 0.07 ± 0.05 in the control group (*p* = 0.21) (Figure 4). For Collagen III, values were 0.10 ± 0.04 in controls and 0.15 ± 0.11 in the stenosis group (*p* = 0.38). Collagen VI showed comparable levels between groups (0.28 ± 0.12 vs. 0.30 ± 0.12, in controls and LSS, respectively, *p* = 0.90), while Collagen XII tended to be lower in the stenosis group (0.06 ± 0.03 in LSS vs. 0.09 ± 0.05 in controls), although without statistical significance (*p* = 0.13) (Figure 4).

### 2.2. Inflammation Assessment

TNF-α was measured as a pro-inflammatory mediator in the two groups of samples; the average concentration was 23.44 ± 25.12 pg/mg in the stenosis group and 13.3 ± 5.06 pg/mg in the control group (*p* = 0.80) (Figure 5A). Moreover, TNF-α levels showed inter-individual variation within the LSS group, with some individuals exhibiting elevated concentrations and others presenting values within the range observed in control tissues. These findings suggest that TNF-α-mediated pro-inflammatory activity is not a consistent feature of LSS, but may characterize only a subset of patients. The scatter plot analysis of TNF-α levels versus ODI scores showed a weak inverse relationship (*r* = −0.18, *p* = 0.53, Figure 5B); TNF-α levels tended to slightly decrease with increasing disability, although the distribution was highly heterogeneous and the association did not reach statistical significance (Figure 5B).

Two patients exhibited higher inflammatory activity: Patient 9 (ODI = 50%) showed the highest TNF-α concentration (71.8 pg/mg), whereas Patient 12 (ODI = 34%) presented a TNF-α level of 43.9 pg/mg (Figure 5). A descriptive comparison of inflammatory and fibrotic profiles within the LSS cohort revealed considerable differences between individual patients. While most samples displayed low-to-moderate TNF-α levels together with increased collagen content, Patients 9 and 12 exhibited elevated TNF-α levels accompanied by relatively high hydroxyproline content (~1.0 and ~0.6 µg/mg, respectively), suggesting that increased inflammatory activity and matrix remodeling may coexist in some individuals.

Conversely, the LSS group showed a significant increase in MMP-2 compared to controls: the mean matrix metalloproteinase-2 (MMP-2) concentration was 18.45 ± 5.15 ng/mg in the stenosis group and 11.63 ± 4.73 ng/mg in the control group, with a difference statistically significant (*p* = 0.0379) also after adjustment for age and BMI using ANCOVA (Figure 6A). Moreover, MMP-2 levels showed a weak positive linear association with disease severity and clinical outcome (*r* = 0.42, *p* = 0.13), with higher ODI scores tending to be associated with greater amount of MMP-2 (Figure 6B).

## 3. Discussion

This study provides the first molecular characterization of TLF in patients with LSS, demonstrating a pattern of active fibrotic remodeling, altered extracellular matrix turnover, and heterogeneous inflammatory activation. Three main findings emerge: increased collagen deposition demonstrated by higher hydroxyproline content, elevated MMP-2 levels, indicating active extracellular matrix remodeling, heterogeneous TNF-α expression, suggesting that increased pro-inflammatory activity may be present only in a subset of LSS patients. Overall, these findings indicate that TLF is an actively remodeled tissue rather than a passive bystander in LSS and may contribute to the complexity and variability of the disease. These data may help to explain why there is still a lack of consensus regarding the best treatment strategy for patients suffering from LSS [4,9,18,19,20] and why a substantial proportion of patients undergo to surgical decompression report unsatisfactory outcomes or require revision surgery [7,14,15,16,17,18].

### 3.1. Collagen Deposition and Remodeling

The most relevant finding of the present study was the significantly increased hydroxyproline content observed in TLF of patients with LSS. Since hydroxyproline is a well-established marker of total collagen content, this result indicates that the thoracolumbar fascia in LSS patients is not simply exposed to degeneration but undergoes an active extracellular matrix remodeling, consistent with fibrotic features and altered tissue homeostasis, potentially reflecting chronic mechanical overload. These molecular findings are consistent with previous ultrasound and MRI studies reporting increased thickness, stiffness, adhesions, and reduced mobility of TLF in subjects with LBP [35,36,37,38,39,40,41,42]. Notably, hydroxyproline content showed a weak inverse relationship with ODI scores. Although collagen accumulation was clearly increased in patients with LSS compared with trauma controls, higher collagen levels were not associated with greater disability. This finding suggests that fascial fibrosis may be a feature of the disease process rather than a direct cause of symptom severity. Clinical disability in LSS is likely influenced by multiple interacting factors, including neural compression, muscle dysfunction, inflammation, and psychosocial variables, which may explain the absence of a statistically significant association between fascial collagen content and ODI scores. Furthermore, it is critical to note that the ODI serves as a comprehensive metric of global clinical disability, which in LSS is predominantly driven by central canal or foraminal nerve root compression and subsequent neurogenic claudication. Therefore, structural and molecular modifications within TLF should be interpreted as a concomitant pathogenic factor or a secondary tissue adaptation, rather than a primary driver that correlates linearly with acute neurological impairment.

Several previous studies have shown that aging is associated with increased stiffness of the intramuscular ECM, which is rich in collagen [44], as well as of the epimysium in rats [45]. Moreover, the thickness of fascial structures has been reported to increase with age [46,47,48]. However, within our study population, age was not associated with collagen concentration in either cohort (Figure 2). Although patients with LSS tended to be older than controls, this difference was not statistically significant. Therefore, while an influence of age on fascial remodeling cannot be completely excluded, our age- and BMI-stratified analyses in the cohort demonstrated higher hydroxyproline levels in the LSS group across all matching age and BMI intervals (Figure 2). Together, these findings support the interpretation that the observed fascial fibrotic remodeling is primarily driven by the underlying LSS pathology rather than age or BMI variables alone, as confirmed by ANCOVA analysis.

Although total collagen content was significantly increased, no significant differences were detected in the individual collagen subtypes. Nevertheless, the observed trends may remain biologically relevant, although they do not allow us to conclude that any specific collagen subtype is responsible for the observed increase in total collagen content. Collagens I and III, the principal components of fibrotic extracellular matrix remodeling, tended to be more abundant in the LSS group, consistent with increased tissue stiffness and altered mechanical properties [49,50,51,52,53,54,55]. Collagen VI, the predominant collagen subtype within TLF and a known regulator of fibroblast activation and fibrotic progression [56,57,58,59], showed comparable levels between groups, suggesting that its role may be predominantly structural rather than reactive role in this context. Conversely, Collagen XII tended to be reduced in the LSS group, potentially reflecting impaired fibril organization and altered extracellular matrix architecture [60].

Overall, these findings suggest a complex reorganization of the fascial extracellular matrix rather than selective overexpression of a single collagen subtype. Larger studies are warranted to clarify the specific contribution of each collagen family to fascial remodeling in LSS.

### 3.2. Inflammatory Activity and Extracellular Matrix Remodeling

Regarding inflammation, differences were observed in the two groups. MMP-2 levels were significantly increased in all the patients with LSS (*p* = 0.0379), suggesting the presence of an active and persistent remodeling process within TLF. Indeed, matrix metalloproteinases are key regulators of extracellular matrix turnover and chronic tissue remodeling.

In contrast to MMP-2, TNF-α levels did not differ significantly between groups.

Although mean TNF-α levels were higher in the LSS group, this difference was driven by a small number of patients with markedly elevated values. Most LSS samples showed TNF-α levels comparable to controls, indicating that increased TNF-α-associated pro-inflammatory activity is not a consistent feature of fascial involvement in LSS. This inter-individual variability highlights that group averages may not fully capture the heterogeneous inflammatory profiles within the LSS population.

Given the pilot nature and limited sample size of our study, these findings should be interpreted with caution and should not considered evidence of distinct molecular patterns. Nevertheless, the observed variability may reflect heterogeneous TLF responses: while most patients showed fibrotic remodeling without marked acute inflammatory activation, a few exhibited concurrent increases in TNF-α and hydroxyproline, suggesting that acute inflammation may coexist with extensive collagen deposition in specific individuals or disease stages. Whether these differences represent distinct stages of the same pathology or patient-specific mechanisms remains unclear. Overall, this molecular heterogeneity highlights the non-uniform nature of fascial involvement in LSS and warrants confirmation in larger longitudinal studies. However, this individual variability may partly explain the inconsistent clinical response to anti-inflammatory therapies reported in the literature [4,18,61,62], as inflammatory mechanisms may not be equally relevant across all patients. Although the limited sample size prevents definitive conclusions, our findings raise the possibility that distinct inflammatory phenotypes may exist within the LSS population. Such heterogeneity may have potential important therapeutic implications for future stratification of patients who may benefit from anti-inflammatory treatments from those who might respond better to rehabilitation approaches targeting fascial mobility and tissue mechanics (Figure 7). However, larger studies are required to validate whether molecular profiles can reliably guide therapeutic decisions.

Furthermore, the data show a weak inverse trend between ODI score and TNF-α levels, suggesting that in LSS, higher disability is not necessarily associated with increased acute inflammatory activity, but rather with lower TNF-α values. This pattern may suggest a predominance of chronic and degenerative tissue changes over acute inflammatory activity, although the limited sample size does not allow the identification of distinct stages of disease progression. However, this interpretation is supported by the weak positive association observed between MMP-2 levels and ODI score. Although the association did not reach statistical significance, it may suggest that higher MMP-2 levels are associated with worse clinical outcomes in LSS. Taken together, the increased hydroxyproline and MMP-2 levels suggest that TLF in LSS is primarily characterized by increased collagen deposition and altered extracellular matrix remodeling. Although acute inflammatory activity was detectable, it was not consistently higher than in control tissues, suggesting that inflammation alone is unlikely to represent the defining feature of fascial involvement in LSS. Importantly, control fascia should not be interpreted as a completely inflammation-free condition, as baseline or trauma-related inflammatory activity may have influences on the measured TNF-α and MMP-2 levels. Therefore, the lack of a significant difference between groups does not exclude inflammatory involvement in LSS, but rather indicates that marked acute inflammatory activation may be limited to a subset of patients. Whether these alterations represent a primary pathogenic mechanism or secondary adaptations to continuous mechanical and inflammatory stress remains unclear. Nevertheless, our findings demonstrate that TLF is not merely a passive connective tissue surrounding the lumbar spine, but an actively remodeled structure that may participate in the pathophysiology of LSS.

### 3.3. Toward a Model of Fascial Involvement in LSS

Overall, our molecular findings provide a biological explanation for the structural alterations previously observed by ultrasound and MRI, suggesting that increased fascial thickness and altered mobility may reflect an underlying fibrotic remodeling process, involving increased collagen deposition, alterations in extracellular matrix organization, and inflammatory activity. Overall, our findings support a model in which chronic mechanical loading associated with lumbar spinal stenosis promotes progressive extracellular matrix remodeling of TLF. This remodeling is characterized by increased collagen accumulation together with persistent matrix turnover, ultimately leading to structural stiffening that may contribute to impaired fascial gliding and persistent low back pain (Figure 8).

### 3.4. Limitations

Some limitations should also be acknowledged. First of all, this cross-sectional design does not allow conclusions regarding causality. In addition, the control group consisted of patients undergoing surgery for acute vertebral trauma, as obtaining fascial biopsies from asymptomatic individuals would raise ethical concerns. Therefore, control tissues may not represent a completely quiescent inflammatory state, and the presence of trauma-related or baseline inflammatory activity cannot be excluded. This aspect should be considered when interpreting the lack of significant differences in TNF-α levels between the LSS and control groups. Although control biopsies were collected within a standardized temporal window after trauma, the limited sample size represents an important constraint. Notably, inflammatory levels in most stenosis samples were comparable to those observed in controls. This does not necessarily indicate the absence of inflammatory processes in LSS, but rather suggests that acute inflammatory activation is not uniformly increased across the LSS population. Indeed, only two patients exhibited higher TNF-α concentrations. Although ANCOVA adjustment for age and BMI supported the persistence of the observed group differences, the limited sample size (*n* = 7 per group) represents an important constraint of this study. A post hoc power analysis indicated a limited statistical power to detect differences in individual collagen subtypes and TNF-α. Therefore, Type II errors cannot be excluded, and non-significant findings should be interpreted with caution. Moreover, although MMP-2 represents a relevant marker of extracellular matrix turnover and remodeling, the assessment of additional molecular factors specifically involved in matrix turnover and chronic inflammatory activity could provide a more comprehensive characterization of the molecular alterations occurring in TLF in LSS. Finally, patients enrolled in the control group were younger and had a lower BMI compared to the cases. This finding may be attributed to the fact that trauma is the primary cause of death among Europeans under 40 years old [63] and obesity increases with age, peaking among people aged between 65 and 74 years old [64]. However, these differences were not statistically significant, (*p* = 0.073 and *p* = 0.053, respectively), and hydroxyproline levels remained consistently higher in the LSS group across age and BMI ranges. Lastly, while the same number of patients was enrolled in each group, the proportion of female participants was lower in the control cohort. However, this finding is consistent with epidemiological evidence showing that males are more frequently involved in traumatic events and road traffic injuries resulting in severe clinical damage [63,64,65].

## 4. Methods

### 4.1. Sample Collection

This study was approved by the Ethics Committee of the Hospital of the University of Padova (approval no. 5473/AO/22, protocol no. AOP2648). All ethical regulations regarding research conducted on human tissues were carefully followed. Patients scheduled for spinal surgery for the treatment of LSS or vertebral fractures at the Orthopaedic Clinic of the University of Padua between January 2025 and November 2025 were invited to participate in the study, and informed consent was obtained from all those who agreed to participate. Inclusion criteria were diagnosis of LSS and vertebral fracture requiring surgery, diagnosis of LSS confirmed by two orthopedic surgeons and patient age between 18 and 80 years old. Individuals suffering from LSS were assigned to the group “cases”; individuals with vertebral fractures were assigned to the group “controls”. Patients undergoing revision surgery or having other surgical indications, patients with unclear diagnosis, patients affected by neoplastic disease of the spine and patients younger than 18 or older than 80 years old were excluded. Diagnosis of LSS was obtained by patient examination conducted by two orthopedic surgeons and by X-ray, CT scan and MRI analysis, according to the most recent guidelines on the topic [4]. Diagnosis of vertebral fracture was obtained by patient examination and by X-ray and CT scans [66,67].

The average age of all participants was 52.1 ± 19.1 years (range, 20–79 years). The control group had a mean age of 43.6 ± 19.3 years (range, 20–73 years), whereas the LSS group had a mean age of 60.6 ± 15.8 years (range, 29–79 years). The mean BMI of the overall cohort was 25.8 ± 4.5 kg/m^2^, with values of 23.7 ± 4.1 kg/m^2^ in the control group and 27.9 ± 4.1 kg/m^2^ in the LSS group.

Moreover, patients suffering from LSS filled out the Oswestry Disability Index questionnaire (ODI) [3,43] the day prior to surgery. During the surgical procedure, a sample of TLF (approximately 0.5 × 2 cm) was collected from each patient between the levels of L1-L3 according to the incision required for the surgical procedure. The sample was placed into phosphate-buffered saline (PBS) and transported to the laboratory within a few hours from collection. It was frozen at −80 °C and then processed before undergoing biochemical analysis.

### 4.2. Collagen Content

The Hydroxyproline Assay Kit (MAK008, Sigma-Aldrich, St. Louis, MO, USA) was used to measure the hydroxyproline content in all the samples, as this molecule is a major component of collagen and largely restricted to it; therefore, measuring hydroxyproline levels can be used as an indicator of collagen content.

The samples were cut into small fragments and homogenized using a mechanical homogenizer in dH_2_O (100 µL/10 mg). For each sample, 100 µL of the homogenate was added to 100 µL of NaOH 10 N for 3 h at 120 °C, and then centrifuged at 10,000× *g* for 3 min. Subsequently, 30 µL of each sample was transferred into 96 microwell plates and subjected to evaporation at 65 °C, to dryness. In each well, 100 µL of oxidation reagent mix (6 µL Chloramine T concentrate + 94 µL oxidation buffer) were added and incubated for 5 min at RT to allow for oxidation of the hydroxyproline molecules. For the development of the colorimetric reaction, 50 µL of Perchloric Acid/Isopropanol Solution were mixed with 50 µL of concentrate DMAB (para-Dimethylaminobenzaldehyde), added in each well and then incubated for 90 min at 60 °C.

The absorbance was measured at 560 nm using the VICTOR-3™ automated microplate reader (Perkin Elmer, Waltham, MA, USA) and converted into µg of hydroxyproline/mg of tissue, based on the standard curve obtained with the collagen standard solution (from 0 to 1 µg/µL).

### 4.3. Immunoblotting for Collagen I, III, VI and XII

Briefly, the frozen samples were thawed, cut with a surgical scalpel, and mechanically digested; total proteins were extracted using RIPA lysis buffer (Thermo Scientific, Waltham, MA, USA) and quantified with the BCA Protein Assay Kit (Thermo Scientific). Equal amounts of proteins (20 µg) were separated on precast polyacrylamide gel with a gradient of 4–25% (Mini-PROTEAN^®^ TGX™ Precast Gels, Bio-Rad, Hercules, CA, USA) and transferred onto polyvinylidene difluoride membranes, PVDF (Bio-Rad). The membranes were incubated with a blocking solution for 1 h at room temperature, and then incubated overnight at 4 °C with the corresponding primary antibodies as follows:

- Anti-Col I in goat (Southern Biotech, Birmingham, AL, USA, 1:1000) in TBS (Tris-buffered saline solution) +5% non-fat dry milk, blocking solution TBS + 5% non-fat dry milk;
- Anti-Col III in rabbit (Abcam, Cambridge, UK, 1:6000) in 0.5% BSA in PBS, blocking solution PBS + BSA 4%;
- Anti-Col VI in rabbit (Proteintech, Rosemont, IL, USA, 1:2000) in PBS + 2% non-fat dry milk, blocking solution PBS + 5% non-fat dry milk;
- Anti-Col XII in mouse (Santa Cruz, Dallas, TX, USA, 1:1000) in 2% BSA in PBS, blocking solution PBS + 5% non-fat dry milk.

After repetitive washes in PBS or TBS, the membranes were incubated, respectively, with goat anti-rabbit–RP (Jackson ImmunoResearch, West Grove, PA, USA, 1:5000), rabbit anti-goat (Jackson ImmunoResearch, 1:12,000) or goat anti-mouse (Jackson ImmunoResearch, 1:5000) antibody for 1 h at room temperature.

After repetitive washes, the immunoreactive reaction was determined by SuperSignal™ West Pico PLUS Chemiluminescent Substrate (Thermo Scientific). The intensity of the bands was measured in ATOM UVITEC (Uvitec, Milan, Italy). Images were analyzed with the Q9 Alliance Software (Uvitec, Milan, Italy) and normalized on the same membrane based on the total protein amount transferred to the membrane, evaluated with Ponceau-S staining and subsequent analysis with ImageJ Software 1.54p (Analyze Gel–Plot Lanes) (freely available at http://rsb.info.nih.gov/ij/, accessed on 2 February 2026) [68]. Each protein (Collagen I, Collagen III, Collagen VI and Collagen XII) was analyzed in all the samples at least in duplicates. The original blots with labels are provided in the Supplementary Materials.

### 4.4. ELISA Immunoassay for Inflammation

Inflammatory activity and extracellular matrix remodeling were assessed in tissue lysates by measuring tumor necrosis factor-alpha (TNF-α) and matrix metalloproteinase-2 (MMP-2), respectively, using an ELISA-based approach.

The human TNF-α ELISA kit (RAB1089, Millipore, Burlington, MA, USA) was used to quantify the levels of TNF-α in tissue lysates. The standard curve was obtained with the Human TNF-α Protein Standard, supplied in the kit. For each sample, a minimum of 2 mg of protein per 1 mL of original lysate was used and diluted 10-fold with 1× Sample Diluent Buffer. A total of 100 µL of each sample were incubated in the Human TNF-alpha Antibody-coated ELISA plate overnight at 4 °C with gentle shaking. After 4 repetitive washes, 100 µL of Biotinylated detection antibody was added in each well for 1 h and then washed. After 45 min of incubation with 100 µL of the HRP–streptavidin solution at RT and subsequent washing, 100 µL of TMB substrate was added (30 min in the dark) to visualize the enzymatic reaction. Then, 50 µL of stop solution was used to stop the reaction and the OD values were immediately measured at 450 nm.

Human matrix metalloproteinase-2 (MMP-2) concentrations were quantified by a sandwich enzyme-linked immunosorbent assay (ELISA) using a commercially available kit (Invitrogen™, Thermo Fisher Scientific, Waltham, MA, USA), following the manufacturer’s protocol. Briefly, 50 μL of incubation buffer followed by 50 μL of samples or serially diluted standards were added to antibody-coated microplate wells, After 2 h of incubation at room temperature, wells were aspirated and washed four times with 1× wash buffer to remove unbound material. A biotin-conjugated anti-human MMP-2 detection antibody (100 μL) was then added to each well, incubated for 1 h, and washed four times, followed by addition of 100 μL of streptavidin–HRP solution and incubation for 30 min with subsequent washing. Colorimetric detection was achieved by adding 100 μL of stabilized tetramethylbenzidine (TMB) substrate and incubating for 30 min in the dark, after which the reaction was terminated with 100 μL of stop solution, producing a color change from blue to yellow. Absorbance was measured at 450 nm.

For both the ELISAs assays, the VICTOR-3™ automated microplate reader was used, (Perkin Elmer, Waltham, MA, USA). TNF-alfa and MMP-2 concentrations were interpolated from the standard curves and corrected for dilution factors.

### 4.5. Statistical Analysis

Statistical analysis was performed using GraphPad Prism 8.4.2 (GraphPad Software Inc., San Diego, CA, USA). All analyses were performed in at least duplicate for each sample. Data are presented as mean ± standard deviation (SD), and a *p*-value < 0.05 was considered statistically significant. Given the limited sample size (*n* = 7 per group), comparisons between the LSS and control groups were performed using the non-parametric Mann–Whitney U test. To assess the linear associations between hydroxyproline content and age, BMI, and ODI, as well as between TNF-α or MMP-2 levels and ODI, Pearson’s correlation coefficient (*r*) and the corresponding *p*-value were calculated. Simple linear regression analysis was also performed, and the coefficient of determination (R^2^) was calculated to quantify the proportion of variability in the dependent variable explained by each predictor.

To assess the potential influence of age and BMI on the observed differences between the LSS and control groups, analysis of covariance (ANCOVA) was performed for hydroxyproline and MMP-2 levels, with group (LSS vs. control) as the main factor and age and BMI as covariates. A post hoc power analysis was also performed to assess the statistical power of the analyses of the individual collagen subtypes and TNF-α.

## 5. Conclusions

To sum up, our results provide the first molecular basis linking previously reported imaging abnormalities with fascial biology and support further investigation of the thoracolumbar fascia as a therapeutic target. TLF should no longer be considered merely a passive anatomical envelope but rather an active connective tissue undergoing extracellular matrix remodeling in LSS. A better understanding of the molecular and structural alterations of TLF may provide a basis for future studies aimed at determining their diagnostic and prognostic relevance and their potential targeted therapeutic implications in patients with LSS.

## Figures and Tables

**Figure 1:**
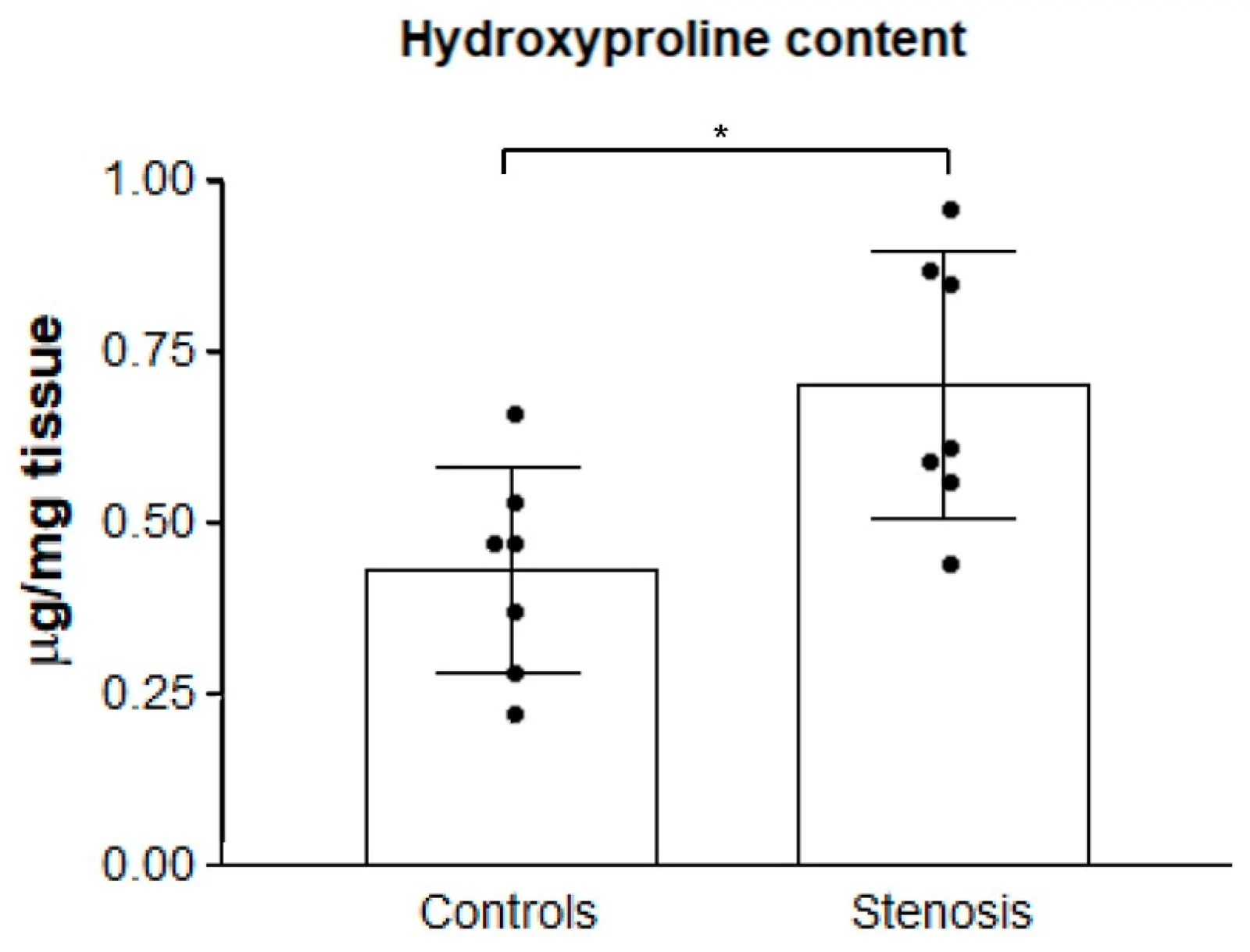
Quantification of hydroxyproline content (µg/mg of tissue) in TLF of LSS and ctrl group. * *p* < 0.05, Mann–Whitney test.

**Figure 2:**
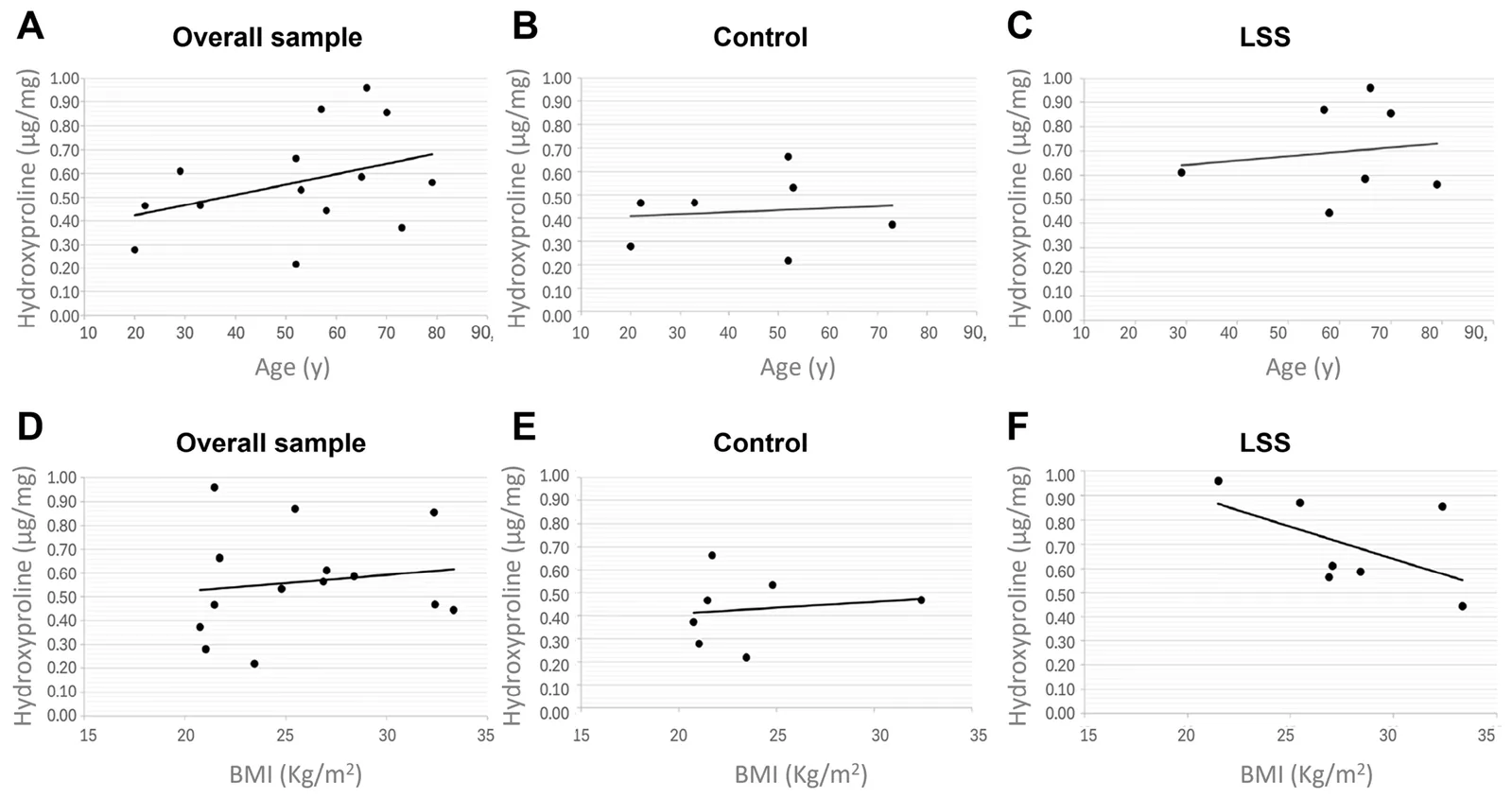
Association between hydroxyproline content and age ((**A**): overall sample, (**B**): control group, (**C**): LSS group) and BMI ((**D**): overall sample, (**E**): control group, (**F**): LSS group). Linear regression: R^2^ = 0.1448 (**A**), 0.0110 (**B**), 0.0216 (**C**), 0.0211 (**D**), 0.0188 (**E**), 0.3080 (**F**). Pearson’s correlation coefficients: *r* = 0.38 (*p* = 0.18) (**A**), *r* = 0.10 (*p* = 0.72) (**B**), *r* = 0.15 (*p* = 0.61) (**C**), *r* = 0.14 (*p* = 0.62) (**D**), *r* = 0.14 (*p* = 0.64) (**E**), *r* = −0.55 (*p* = 0.04) (**F**).

**Figure 3:**
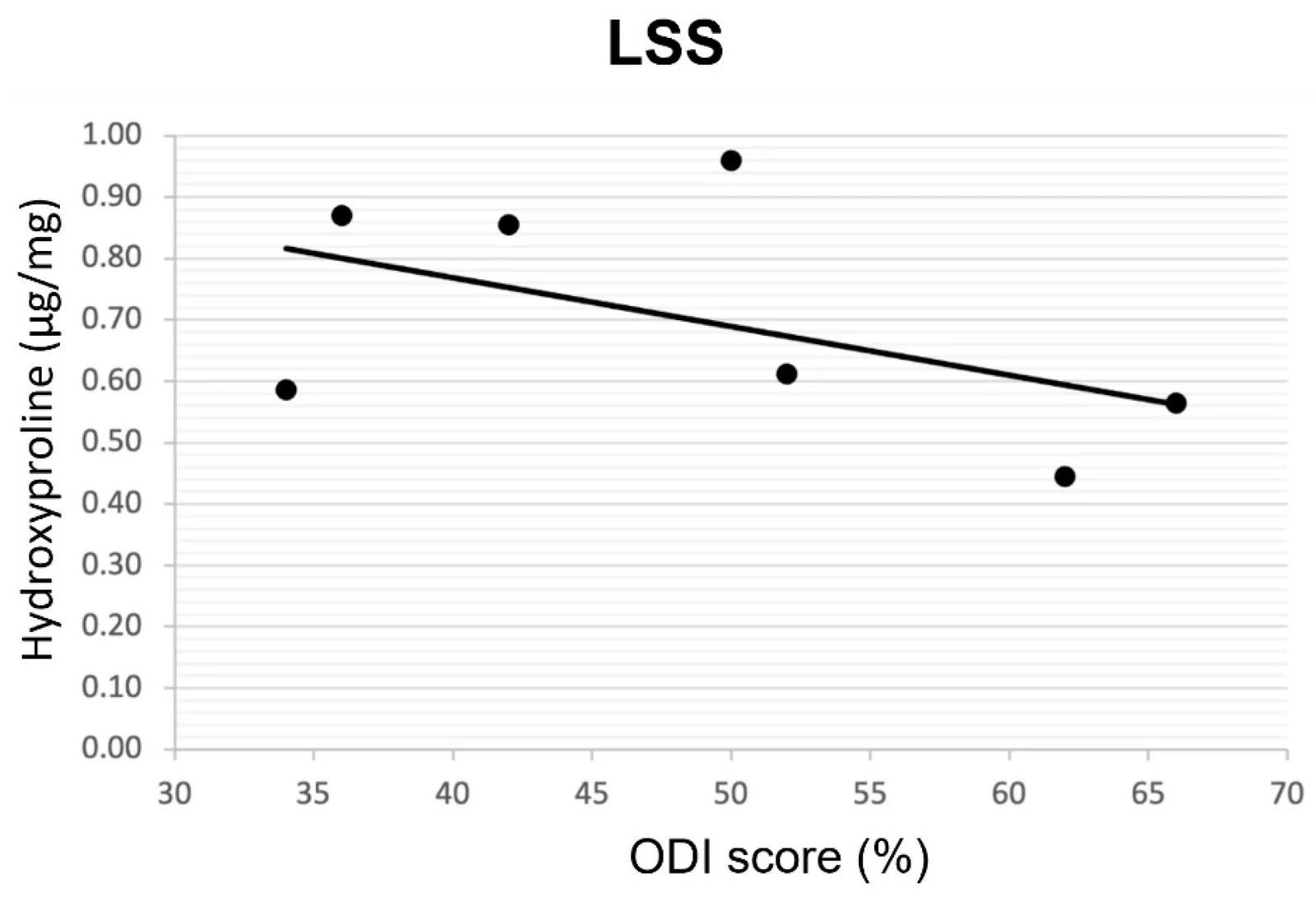
Linear regression analysis between hydroxyproline content and ODI score in LSS patients. Linear regression: R^2^ = 0.2561. Pearson’s correlation coefficients: *r* = −0.51 (*p* = 0.06).

**Figure 4:**
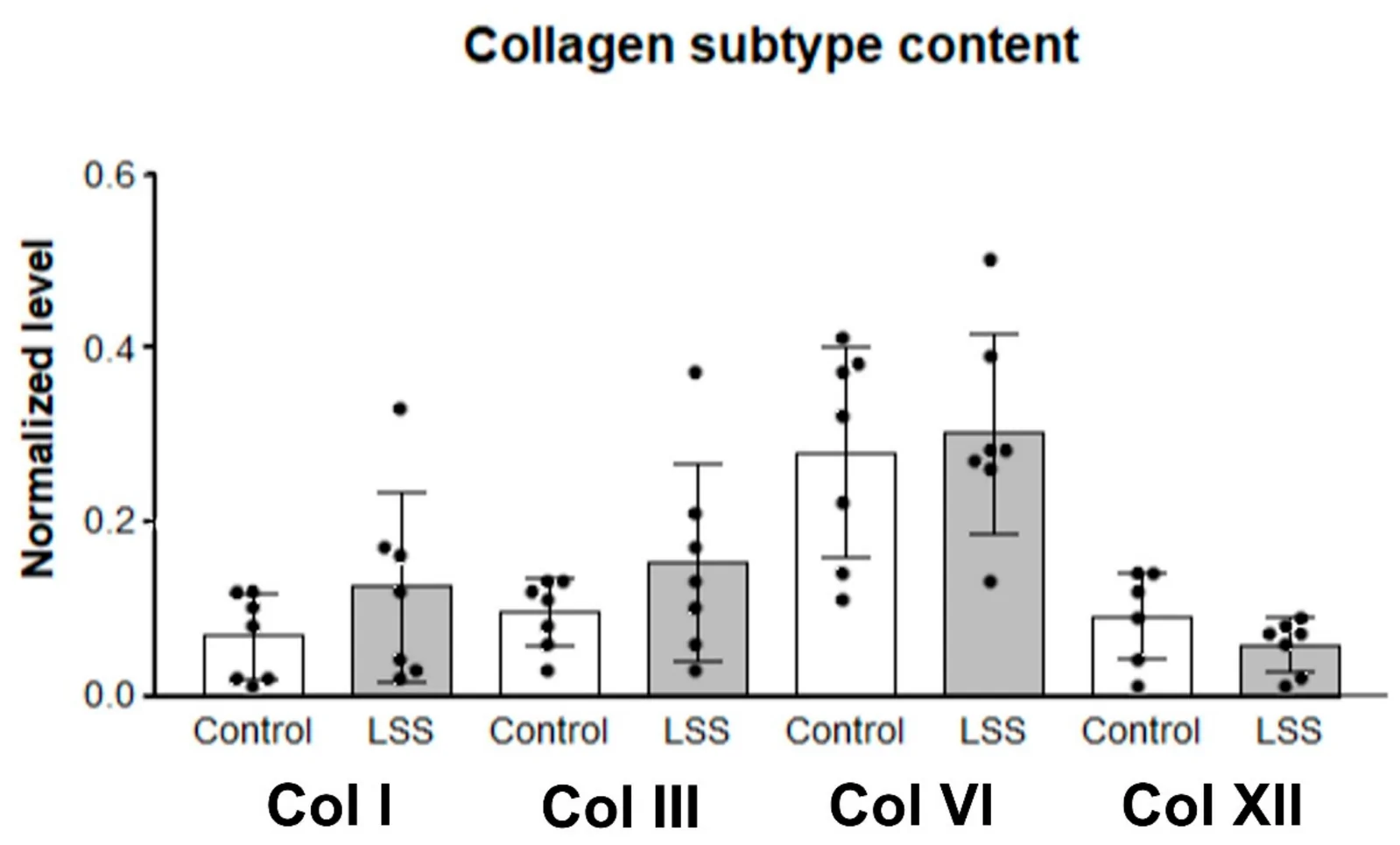
Comparison of normalized collagen subtype content (Collagen I, Collagen III, Collagen VI, and Collagen XII) between lumbar spinal stenosis (LSS) and control groups. Data are presented as mean ± standard deviation. No statistically significant differences were observed between groups for any collagen subtype (all *p* > 0.05).

**Figure 5:**
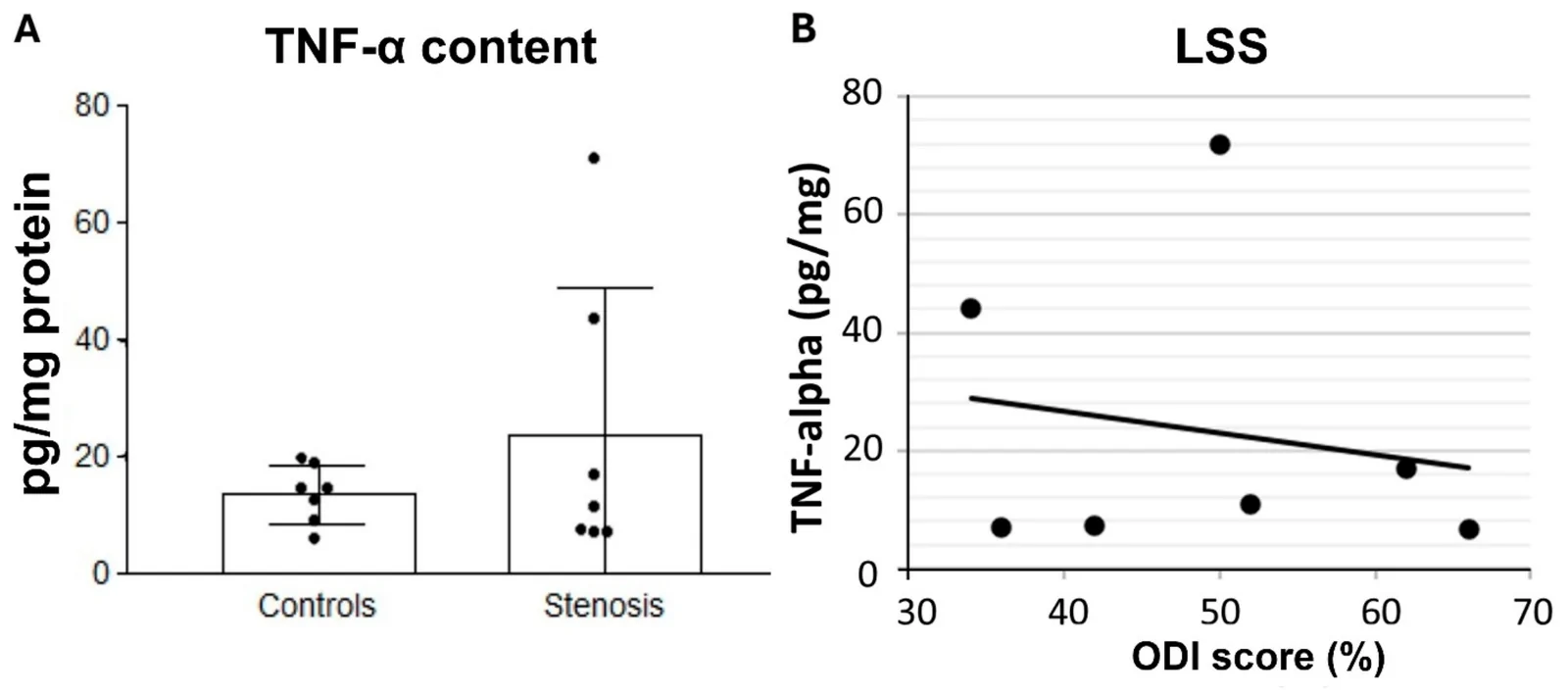
(**A**): Mean values of TNF-α in control and stenosis groups. (**B**): Linear regression analysis of TNF-α values in relation to ODI scores in the LSS group. Linear regression: R^2^ = 0.0334. Pearson’s correlation coefficients: *r* = −0.18 (*p* = 0.53) (**B**).

**Figure 6:**
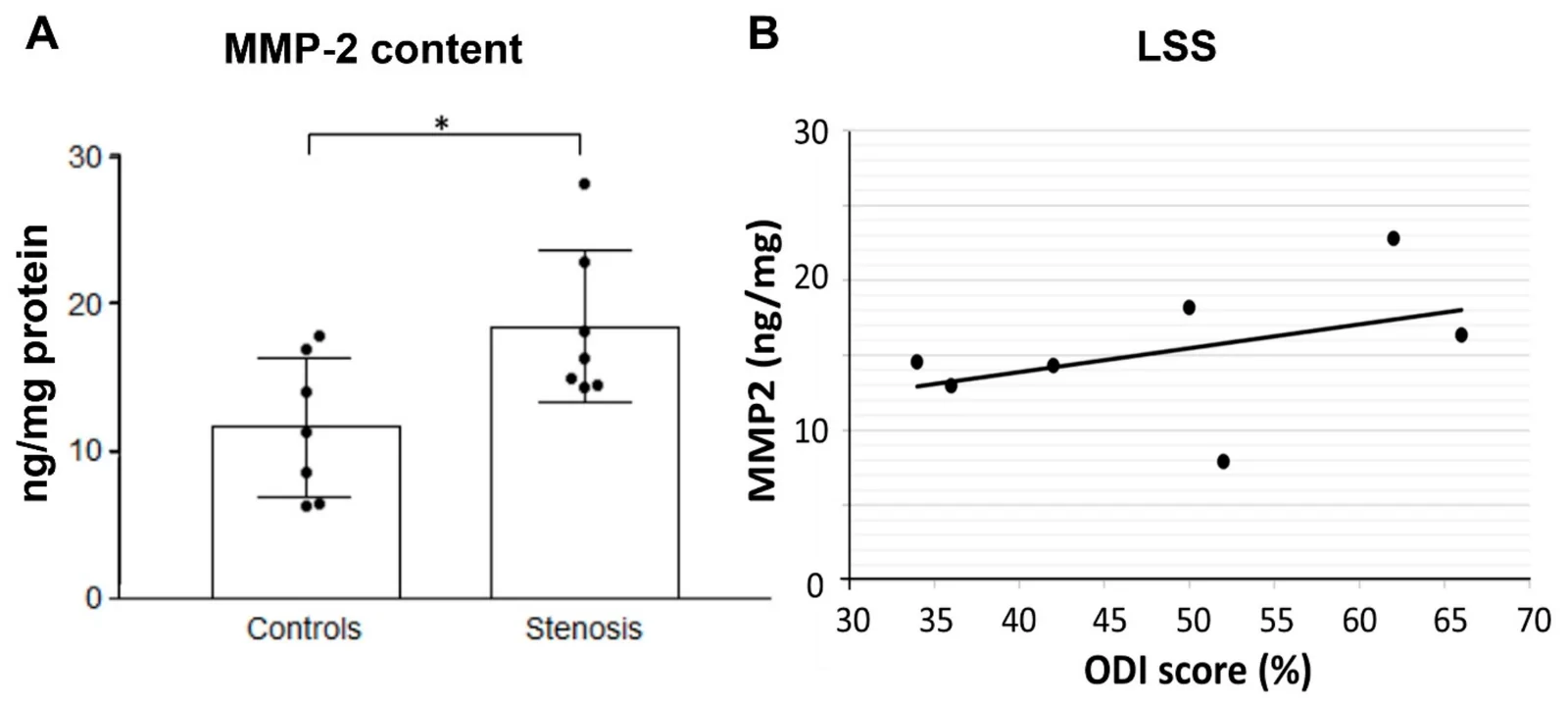
(**A**): Mean values of MMP-2 in controls and stenosis groups. * *p* < 0.05. (**B**): Linear regression analysis of MMP-2 values in relation to ODI scores in the LSS group. Linear regression: R^2^ = 0.1799. Pearson’s correlation coefficient: *r* = 0.42, *p* = 0.13 (**B**).

**Figure 7:**
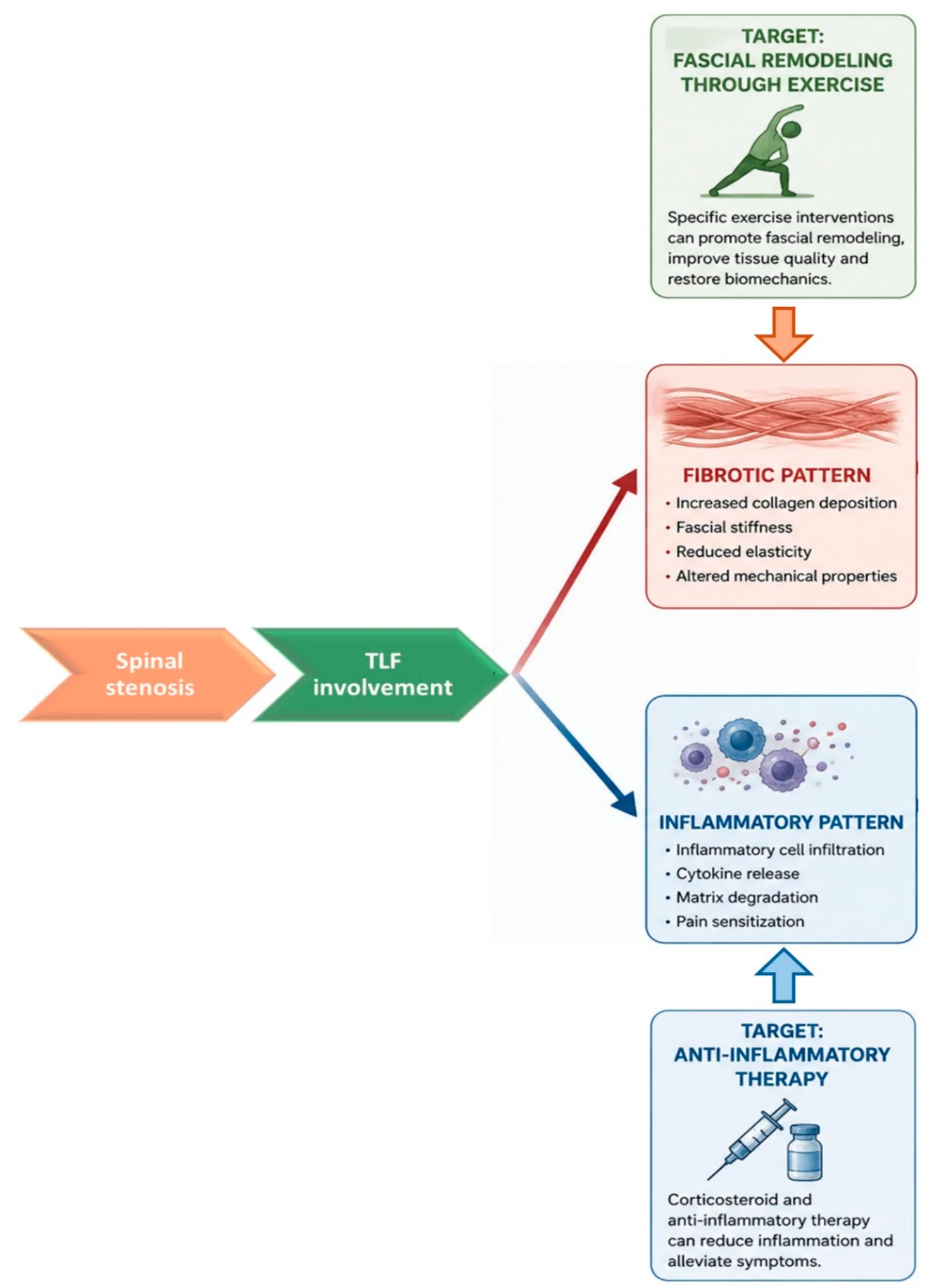
Hypothetical model of clinical stratification based on preliminary TLF molecular alterations.

**Figure 8:**
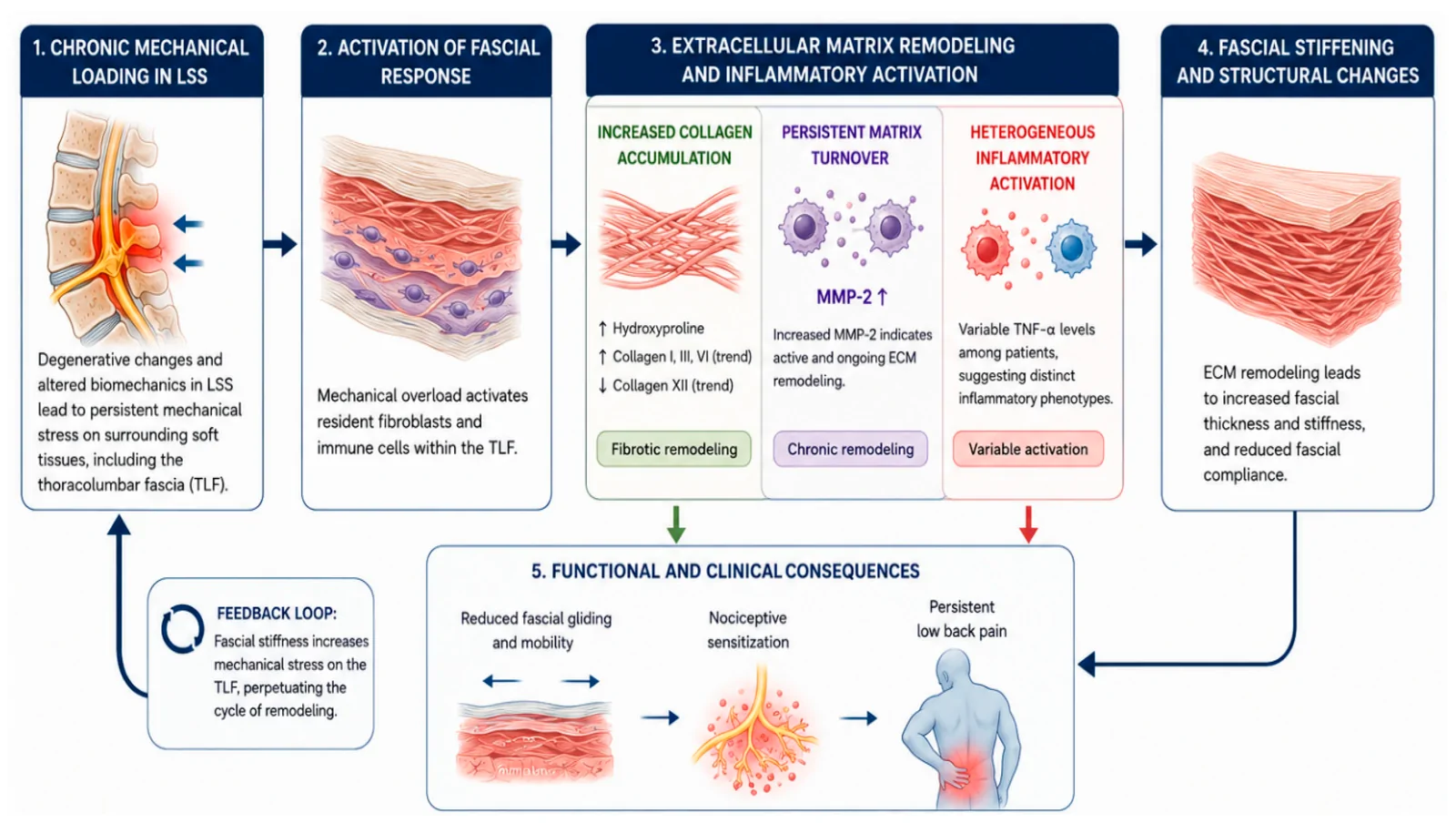
Proposed hypothetical model of TLF remodeling under chronic mechanical stress in lumbar spinal stenosis.

**Table 1:** Demographic information for the control trauma group and LSS group.

	Sex	Age (y)	Weight (kg)	Height (m)	BMI (kg/m^2^)	ODI (%)
**Controls**						
**1**	M	73.44	73	1.81	20.76	NA
**2**	M	53.35	70	1.68	24.80	NA
**3**	M	20.36	72	1.85	21.04	NA
**4**	M	33.28	105	1.80	32.41	NA
**5**	F	22.57	65	1.74	21.47	NA
**6**	M	52.03	65	1.73	21.72	NA
**7**	M	52.72	76	1.80	23.46	NA
***Total 7***	*6M-1F*	*43.9 ± 19.3*	*75.1 ± 13.8*	*1.77 ± 0.06*	*23.7 ± 4.1*	*--*
**LSS**						
**8**	M	57.01	78	1.75	25.47	36
**9**	F	66.80	65	1.74	21.47	50
**10**	F	70.31	86	1.63	32.37	42
**11**	M	29.12	80	1.72	27.04	52
**12**	M	65.78	85	1.73	28.40	34
**13**	M	79.79	89	1.82	26.87	66
**14**	F	58.25	75	1.50	33.33	62
***Total 7***	*4M-3F*	*61.0 ± 16.0*	*79.7 ± 8.1*	*1.70 ± 0.1*	*27.9 ± 4.1*	*48.8 ± 12.3*

## Data Availability

The original contributions presented in this study are included in the article. Further inquiries can be directed to the corresponding author.

## Supplementary Materials

The following supporting information can be downloaded at https://www.mdpi.com/article/10.3390/ijms27198562/s1.

## References

[1] Lai M.K.L., Cheung P.W.H., Cheung J.P.Y. (2020). A systematic review of developmental lumbar spinal stenosis. Eur. Spine J..

[2] Botwin K., Brown L.A., Fishman M., Rao S. (2007). Fluoroscopically guided caudal epidural steroid injections in degenerative lumbar spine stenosis. Pain Physician.

[3] Watters W.C., Baisden J., Gilbert T.J., Kreiner S., Resnick D.K., Bono C.M., Ghiselli G., Heggeness M.H., Mazanec D.J., O’Neill C. (2008). Degenerative lumbar spinal stenosis: An evidence-based clinical guideline for the diagnosis and treatment of degenerative lumbar spinal stenosis. Spine J..

[4] Kreiner D.S., Shaffer W.O., Baisden J.L., Gilbert T.J., Summers J.T., Toton J.F., Hwang S.W., Mendel R.C., Reitman C.A. (2013). An evidence-based clinical guideline for the diagnosis and treatment of degenerative lumbar spinal stenosis (update). Spine J..

[5] Deyo R.A., Weinstein J.N. (2001). Low Back Pain. N. Engl. J. Med..

[6] Hoy D., March L., Brooks P., Blyth F., Woolf A., Bain C., Williams G., Smith E., Vos T., Barendregt J. (2014). The global burden of low back pain: Estimates from the Global Burden of Disease 2010 study. Ann. Rheum. Dis..

[7] Melcher C., Paulus A.C., Roßbach B.P., Gülecyüz M.F., Birkenmaier C., von Schulze-Pellengahr C., Teske W., Wegener B. (2022). Lumbar spinal stenosis—Surgical outcome and the odds of revision-surgery: Is it all due to the surgeon?. Technol. Health Care.

[8] Zileli M., Crostelli M., Grimaldi M., Mazza O., Anania C., Fornari M., Costa F. (2020). Natural Course and Diagnosis of Lumbar Spinal Stenosis: WFNS Spine Committee Recommendations. World Neurosurg. X.

[9] Zaina F., Tomkins-Lane C., Carragee E., Negrini S. (2016). Surgical versus non-surgical treatment for lumbar spinal stenosis. Cochrane Database Syst. Rev..

[10] Malmivaara A., Slätis P., Heliövaara M., Sainio P., Kinnunen H., Kankare J., Dalin-Hirvonen N., Seitsalo S., Herno A., Kortekangas P. (2007). Surgical or Nonoperative Treatment for Lumbar Spinal Stenosis?: A Randomized Controlled Trial. Spine.

[11] Athiviraham A., Yen D. (2007). Is Spinal Stenosis Better Treated Surgically or Nonsurgically?. Clin. Orthop..

[12] Weinstein J.N., Tosteson T.D., Lurie J.D., Tosteson A., Blood E., Herkowitz H., Cammisa F., Albert T., Boden S.D., Hilibrand A. (2010). Surgical Versus Nonoperative Treatment for Lumbar Spinal Stenosis Four-Year Results of the Spine Patient Outcomes Research Trial. Spine.

[13] Lurie J., Tomkins-Lane C. (2016). Management of lumbar spinal stenosis. BMJ.

[14] Schär R.T., Kiebach S., Raabe A., Ulrich C.T. (2019). Reoperation Rate After Microsurgical Uni- or Bilateral Laminotomy for Lumbar Spinal Stenosis With and Without Low-grade Spondylolisthesis: What do Preoperative Radiographic Parameters Tell Us?. Spine.

[15] Brodke D.S., Annis P., Lawrence B.D., Woodbury A.M., Daubs M.D. (2013). Reoperation and Revision Rates of 3 Surgical Treatment Methods for Lumbar Stenosis Associated with Degenerative Scoliosis and Spondylolisthesis. Spine.

[16] Kelleher M.O., Timlin M., Persaud O., Rampersaud Y.R. (2010). Success and Failure of Minimally Invasive Decompression for Focal Lumbar Spinal Stenosis in Patients With and Without Deformity. Spine.

[17] Modhia U., Takemoto S., Braid-Forbes M.J., Weber M., Berven S.H. (2013). Readmission rates after decompression surgery in patients with lumbar spinal stenosis among Medicare beneficiaries. Spine.

[18] Bussières A., Cancelliere C., Ammendolia C., Comer C.M., Al Zoubi F., Châtillon C.-E., Chernish G., Cox J.M., Gliedt J.A., Haskett D. (2021). Non-Surgical Interventions for Lumbar Spinal Stenosis Leading to Neurogenic Claudication: A Clinical Practice Guideline. J. Pain.

[19] Alimi M., Hofstetter C.P., Pyo S.Y., Paulo D., Härtl R. (2015). Minimally invasive laminectomy for lumbar spinal stenosis in patients with and without preoperative spondylolisthesis: Clinical outcome and reoperation rates. J. Neurosurg. Spine.

[20] Chang Y., Singer D.E., Wu Y.A., Keller R.B., Atlas S.J. (2005). The effect of surgical and nonsurgical treatment on longitudinal outcomes of lumbar spinal stenosis over 10 years. J. Am. Geriatr. Soc..

[21] Minamide A., Yoshida M., Maio K. (2013). The natural clinical course of lumbar spinal stenosis: A longitudinal cohort study over a minimum of 10years. J. Orthop. Sci..

[22] Stecco C., Pratt R., Nemetz L.D., Schleip R., Stecco A., Theise N.D. (2025). Towards a comprehensive definition of the human fascial system. J. Anat..

[23] Willard F.H., Vleeming A., Schuenke M.D., Danneels L., Schleip R. (2012). The thoracolumbar fascia: Anatomy, function and clinical considerations. J. Anat..

[24] Corey S.M., Vizzard M.A., Badger G.J., Langevin H.M. (2011). Sensory Innervation of the Nonspecialized Connective Tissues in the Low Back of the Rat. Cells Tissues Organs.

[25] Tesarz J., Hoheisel U., Wiedenhöfer B., Mense S. (2011). Sensory innervation of the thoracolumbar fascia in rats and humans. Neuroscience.

[26] Schilder A., Hoheisel U., Magerl W., Benrath J., Klein T., Treede R.D. (2014). Sensory findings after stimulation of the thoracolumbar fascia with hypertonic saline suggest its contribution to low back pain. Pain.

[27] Hoheisell U., Taguchil T., Treedel R., Mensel S. (2011). Nociceptive input from the rat thoracolumbar fascia to lumbar dorsal horn neurones. Eur. J. Pain.

[28] Gibson W., Arendt-Nielsen L., Taguchi T., Mizumura K., Graven-Nielsen T. (2009). Increased pain from muscle fascia following eccentric exercise: Animal and human findings. Exp. Brain Res..

[29] Langevin H.M., Sherman K.J. (2007). Pathophysiological model for chronic low back pain integrating connective tissue and nervous system mechanisms. Med. Hypotheses.

[30] Fede C., Petrelli L., Guidolin D., Porzionato A., Pirri C., Fan C., De Caro R., Stecco C. (2021). Evidence of a new hidden neural network into deep fasciae. Sci. Rep..

[31] Mense S., Hoheisel U. (2016). Evidence for the existence of nociceptors in rat thoracolumbar fascia. J. Bodyw. Mov. Ther..

[32] Kondrup F., Gaudreault N., Venne G. (2022). The deep fascia and its role in chronic pain and pathological conditions: A review. Clin. Anat..

[33] Wilke J., Schleip R., Klingler W., Stecco C. (2017). The Lumbodorsal Fascia as a Potential Source of Low Back Pain: A Narrative Review. BioMed. Res. Int..

[34] Casato G., Stecco C., Busin R. (2019). Role of fasciae in nonspecific low back pain. Eur. J. Transl. Myol..

[35] Pirri C., Pirri N., Guidolin D., Macchi V., Porzionato A., De Caro R., Stecco C. (2023). Ultrasound Imaging of Thoracolumbar Fascia Thickness: Chronic Non-Specific Lower Back Pain versus Healthy Subjects; A Sign of a “Frozen Back”?. Diagnostics.

[36] Langevin H.M., Bishop J., Maple R., Badger G.J., Fox J.R. (2018). Effect of Stretching on Thoracolumbar Fascia Injury and Movement Restriction in a Porcine Model. Am. J. Phys. Med. Rehabil..

[37] Tomita N., Destrempes F., Cardinal M.-H.R., Boulanger A., Gaudreault N., Cloutier G. (2025). Thoracolumbar Fascia Microstructure and Low Back Pain: Insights from Quantitative Ultrasound Homodyned K-Distribution Statistical Modeling. Ultrasound Med. Biol..

[38] Tomita N., Roy-Cardinal M.-H., Chayer B., Daher S., Attiya A., Boulanger A., Gaudreault N., Cloutier G., Bureau N.J. (2025). Thoracolumbar fascia ultrasound shear strain differs between low back pain and asymptomatic individuals: Expanding the evidence. Insights Imaging.

[39] Langevin H.M., Stevens-Tuttle D., Fox J.R., Badger G.J., Bouffard N.A., Krag M.H., Wu J., Henry S.M. (2009). Ultrasound evidence of altered lumbar connective tissue structure in human subjects with chronic low back pain. BMC Musculoskelet. Disord..

[40] Langevin H.M., Fox J.R., Koptiuch C., Badger G.J., Naumann A.C.G., Bouffard N.A., Konofagou E.E., Lee W.-N., Triano J.J., Henry S.M. (2011). Reduced thoracolumbar fascia shear strain in human chronic low back pain. BMC Musculoskelet. Disord..

[41] Adamietz B., Schönberg S.O., Reiser M., Uder M., Frank A., Strecker R., Weiß C., Heiss R. (2021). Visualization of the epimysium and fascia thoracolumbalis at the lumbar spine using MRI. Radiologe.

[42] Ranger T.A., Teichtahl A.J., Cicuttini F.M., Wang Y., Wluka A.E., O’sullivan R., Jones G., Urquhart D.M. (2016). Shorter Lumbar Paraspinal Fascia Is Associated with High Intensity Low Back Pain and Disability. Spine.

[43] Fairbank J.C.T., Pynsent P.B. (2022). The Oswestry Disability Index. Spine.

[44] Kjær M. (2004). Role of Extracellular Matrix in Adaptation of Tendon and Skeletal Muscle to Mechanical Loading. Physiol. Rev..

[45] Gao Y., Kostrominova T.Y., Faulkner J.A., Wineman A.S. (2008). Age-related changes in the mechanical properties of the epimysium in skeletal muscles of rats. J. Biomech..

[46] Wilke J., Macchi V., De Caro R., Stecco C. (2019). Fascia thickness, aging and flexibility: Is there an association?. J. Anat..

[47] Pirri C., Pirri N., Guidolin D., Macchi V., De Caro R., Stecco C. (2022). Ultrasound Imaging of the Superficial Fascia in the Upper Limb: Arm and Forearm. Diagnostics.

[48] Zügel M., Maganaris C.N., Wilke J., Jurkat-Rott K., Klingler W., Wearing S.C., Findley T., Barbe M.F., Steinacker J.M., Vleeming A. (2018). Fascial tissue research in sports medicine: From molecules to tissue adaptation, injury and diagnostics: Consensus statement. Br. J. Sports Med..

[49] Gillies A.R., Lieber R.L. (2011). Structure and function of the skeletal muscle extracellular matrix. Muscle Nerve.

[50] Purslow P.P. (2020). The Structure and Role of Intramuscular Connective Tissue in Muscle Function. Front. Physiol..

[51] Booth C.M., Cortina-Borja M.J.F., Theologis T.N. (2001). Collagen accumulation in muscles of children with cerebral palsy and correlation with severity of spasticity. Dev. Med. Child Neurol..

[52] Hillege M., Galli Caro R., Offringa C., De Wit G., Jaspers R., Hoogaars W. (2020). TGF-β Regulates Collagen Type I Expression in Myoblasts and Myotubes via Transient Ctgf and Fgf-2 Expression. Cells.

[53] Klingberg F., Hinz B., White E.S. (2013). The myofibroblast matrix: Implications for tissue repair and fibrosis. J. Pathol..

[54] Wynn T. (2008). Cellular and molecular mechanisms of fibrosis. J. Pathol..

[55] Frantz C., Stewart K.M., Weaver V.M. (2010). The extracellular matrix at a glance. J. Cell Sci..

[56] Naugle J.E., Olson E.R., Zhang X., Mase S.E., Pilati C.F., Maron M.B., Folkesson H.G., Horne W.I., Doane K.J., Meszaros J.G. (2006). Type VI collagen induces cardiac myofibroblast differentiation: Implications for postinfarction remodeling. Am. J. Physiol.-Heart Circ. Physiol..

[57] Theocharidis G., Drymoussi Z., Kao A.P., Barber A.H., Lee D.A., Braun K.M., Connelly J.T. (2016). Type VI Collagen Regulates Dermal Matrix Assembly and Fibroblast Motility. J. Investig. Dermatol..

[58] Williams L.M., McCann F.E., Cabrita M.A., Layton T., Cribbs A., Knezevic B., Fang H., Knight J., Zhang M., Fischer R. (2020). Identifying collagen VI as a target of fibrotic diseases regulated by CREBBP/EP300. Proc. Natl. Acad. Sci. USA.

[59] Fede C., Clair C., Petrelli L., Sun Y., Biz C., Angelini A., Ruggieri P., Stecco C. (2026). Redefining the Collagen Composition of Human Fasciae: Emerging Collagen Types and Structural Heterogeneity. Int. J. Mol. Sci..

[60] Fung A.K., Weiss S.N., Nuss C.A., Yen W., Volk S.W., Dyment N.A., Soslowsky L.J. (2025). Collagen XII Plays a More Prominent Cell-Mediated Role in Tendon Organization Compared to Matrix Assembly During Postnatal Development. FASEB J..

[61] Webster L.R., Markman J. (2014). Medical management of chronic low back pain: Efficacy and outcomes. Neuromodul. J. Int. Neuromodul. Soc..

[62] Rousing R., Jensen R.K., Fruensgaard S., Strøm J., Brøgger H.A., Degn J.D.M., Andersen M.Ø. (2019). Danish national clinical guidelines for surgical and nonsurgical treatment of patients with lumbar spinal stenosis. Eur. Spine J..

[63] Halvachizadeh S., Mariani D., Pfeifer R. (2025). Impact of trauma on society. Eur. J. Trauma Emerg. Surg..

[64] Ng M., Gakidou E., Lo J., Abate Y.H., Abbafati C., Abbas N., Abbasian M., ElHafeez S.A., Abdel-Rahman W.M., Abd-Elsalam S. (2025). Global, regional, and national prevalence of adult overweight and obesity, 1990–2021, with forecasts to 2050: A forecasting study for the Global Burden of Disease Study 2021. Lancet.

[65] Sorenson S.B. (2011). Gender Disparities in Injury Mortality: Consistent, Persistent, and Larger Than You’d Think. Am. J. Public Health.

[66] Kendler D., Bauer D., Davison K., Dian L., Hanley D., Harris S., McClung M., Miller P., Schousboe J., Yuen C. (2016). Vertebral Fractures: Clinical Importance and Management. Am. J. Med..

[67] Vu C., Gendelberg D. (2020). Classifications in Brief: AO Thoracolumbar Classification System. Clin. Orthop..

[68] Sander H., Wallace S., Plouse R., Tiwari S., Gomes A.V. (2019). Ponceau S waste: Ponceau S staining for total protein normalization. Anal. Biochem..

