# Supplementary material for: Thoracolumbar Fascia Remodeling in Lumbar Spinal Stenosis: Translational Orthopedic Perspectives
Source: Int J Mol Sci. 2026 Sep 25;27(19):8562. doi: 10.3390/ijms27198562 (PMC13665421; doi:10.3390/ijms27198562)
Supplement: Supplementary file 1 [file ijms-27-08562-s001.zip › ijms-4514898-supplementary.pdf]

PONCEAU-S

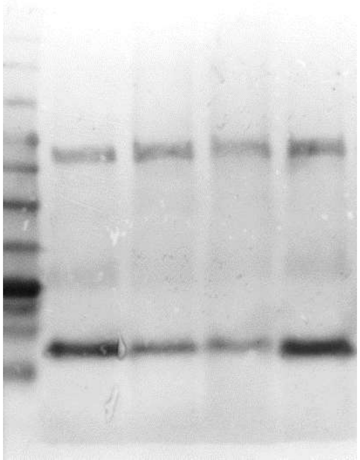

COL 1

1. 2. 3. 4.

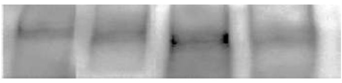

1. Stenosis
2. Stenosis
3. Control
4. Control

COL 6

1. 2. 3. 4.

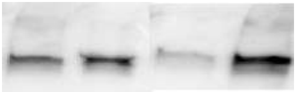

1. Stenosis
2. Stenosis
3. Control
4. Control

COL 3

1. 2. 3. 4.

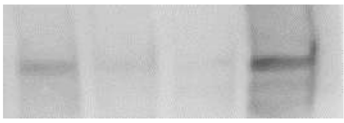

1. Stenosis
2. Stenosis
3. Control
4. Control

COL 12

1. 2. 3. 4.

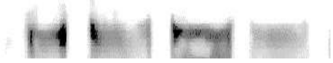

1. Stenosis
2. Stenosis
3. Control
4. Control
